# GP-support by means of AGnES-practice assistants and the use of telecare devices in a sparsely populated region in Northern Germany – proof of concept

**DOI:** 10.1186/1471-2296-10-44

**Published:** 2009-06-19

**Authors:** Neeltje van den Berg, Thomas Fiß, Claudia Meinke, Romy Heymann, Sibylle Scriba, Wolfgang Hoffmann

**Affiliations:** 1 Institute for Community Medicine, University of Greifswald, Ellernholzstraße 1-2, 17487 Greifswald, Germany; 2 Ministry of Health of the Federal State of Mecklenburg-Western Pomerania, Werderstraße 124, 19055 Schwerin, Germany

## Abstract

**Background:**

In many rural regions in Germany, the proportion of the elderly population increases rapidly. Simultaneously, about one-third of the presently active GPs will retire until 2010. Often it is difficult to find successors for vacant GP-practices. These regions require innovative concepts to avoid the imminent shortage in primary health care.

The AGnES-concept comprises the delegation of GP-home visits to qualified AGnES-practice assistants (AGnES: GP-supporting, community-based, e-health-assisted, systemic intervention). Main objectives were the assessment of the acceptance of the AGnES-concept by the participating GPs, patients, and AGnES-practice assistants, the kind of delegated tasks, and the feasibility of home telecare in a GP-practice.

**Methods:**

In this paper, we report first results of the implementation of this concept in regular GP-practices, conducted November 2005 – March 2007 on the Island of Rügen, Mecklenburg-Western Pomerania, Germany. This study was meant as a proof of concept.

The GP delegated routine home-visits to qualified practice employees (here: registered nurses). Eligible patients were provided with telecare-devices to monitor disease-related physiological values.

All delegated tasks, modules conducted and questionnaire responses were documented. The participating patients were asked for their acceptance based on standardized questionnaires. The GPs and AGnES-practice assistants were asked for their judgement about different project components, the quality of health care provision and the competences of the AGnES-practice assistants.

**Results:**

550 home visits were conducted. 105 patients, two GPs and three AGnES-practice assistants (all registered nurses) participated in the project. 48 patients used telecare-devices to monitor health parameters. 87.4% of the patients accepted AGnES-care as comparable to common GP-care. In the course of the project, the GPs delegated an increasing number of both monitoring and interventional tasks to the AGnES-practice assistants. The GPs agreed that delegating tasks to a qualified practice assistant relieves them in their daily work.

**Conclusion:**

A part of the GPs home visits can be delegated to AGnES-practice assistants to support GPs in regions with an imminent or already existing undersupply in primary care. The project triggered discussions among the institutions involved in the German healthcare system and supported a reconciliation of the respective competences of physicians and other medical professions.

## Background

The Federal State of Mecklenburg-Western Pomerania is predominantly rural. Due to the low number of births, the migration of mostly young people to the Western parts of Germany and the increasing life expectancy, the percentage of people of 65 years or older in the population increases rapidly from 10.6% in 1989 to expected 26.5% in 2020 [1,2].

A major consequence of ageing in a population is the increase of age-specific diseases.

Table 1 shows the resulting trend of incidences and prevalences for selected diseases. The consecutive increase of the need for medical care will outweigh the overall decrease of the population in Mecklenburg-Western Pomerania [3].

**Table 1:** Prognosis of the number of cases of some age-specific diseases in Mecklenburg-Western Pomerania in 2012 and 2020, reference: 2002. (Source: (3))

	2012	2020
Diabetes Type II (prevalent cases)	+13.2%	+9.9%
Myocardial infarction (incident cases)	+27.5%	+40.9%
Dementia (prevalent cases)	+40.4%	+67.2%

At the same time, there is a continuous decline of the number of general practitioners (GPs) in the rural parts of Mecklenburg-Western Pomerania, mostly due to their age structure. Presently, about 30% of the GPs in Mecklenburg-Western Pomerania are older than 60 years [4]. As it is difficult to replace retired GPs in rural areas by young physicians, a shortage of GPs is imminent for major parts of Mecklenburg-Western Pomerania.

The German primary care system is physician-centered. GPs run their practices based on mandatory contracts with the Association of Statutory Health Insurance Physicians of Mecklenburg-West Pomerania (= association of physicians with the right to reimburse with the statutory health insurances).

In Germany, the role of other medical professions than physicians in the primary care system is limited. There is a strict distinction between activities and competences of physicians and other professions, e.g. nurses or physician's assistants (practice nurses). In particular, they are not allowed to conduct initial patient-contacts, to diagnose, to initiate a therapy or to prescribe medication. Physician's assistants in GP-practices are mainly responsible for administrative tasks [5].

The GP can choose to delegate certain activities including home visits under certain restrictions to qualified practice employees (nurses or physician's assistants). Liability issues and insufficient reimbursement for home visits by practice employees have limited the use of this option.

The AGnES-concept (AGnES is a German abbreviation: GP-relieving, community-based, e-health-assisted, systemic intervention) was developed to extend this possibility of delegation and make it to a structural part of primary care, especially for sparsely populated regions with an imminent or already existing undersupply with GPs. The AGnES-concept rests on the delegation of original physician-activities, especially home visits, to qualified medical practice personnel, e.g. registered nurses or physician's assistants, here all called AGnES-practice assistants. The AGnES-concept should support GPs and enable them to assure medical care for more patients in a larger area.

There are more examples for successful projects using this strategy, but implementing an innovation into the daily routine of a GP-practice reveals a lot of organizational, logistical and structural details that can't be planned in advance [6,7]. On the other side, only the implementation of an innovation into the health care system allows for data collection and analysis to evaluate the concept [8].

Although the idea for the AGnES-concept originated from the federal state government, the development happened in strong cooperation with the participating GP-practices. Involving practice-teams directly into the research activities was a main strategy of the project.

The implementation e-health in form of home telecare was evaluated as a further option for supporting GPs. In the ageing societies of almost all western countries, the need for regular monitoring of essential physiological parameters and the increasing demand for more safety in the home situation will substantially foster the use of different types of home telecare [9].

Telecare is already used in different areas of medical care and pilot studies have been successfully evaluated for efficacy and – in some instances – also for efficiency. Most studies refer to particular telemedical devices used for selected diseases in a hospital setting. Examples are the implementation of telemedical ECG-devices to monitor cardio-vascular diseases [10,11] or the monitoring of blood glucose levels for diabetes patients [12].

In general practices, home telecare has been applied primarily in remote, sparsely populated regions. First examples of video consulting as allowing consultations with the GP or even email communication between patient and GP have been evaluated with positive results [13-15].

In three short field phases, meant as a proof of concept, qualified AGnES-practice assistants (here: registered nurses) have conducted home visits with older, multimorbid patients with chronic diseases and reduced mobility. To be visited by an AGnES-practice assistant, did not necessarily mean that the GP discontinued his own home-visits to these patients. The GP was free to decide about the frequency of the home-visits, both of his own and of the AGnES-practice assistant.

Optionally, home telecare could be used to monitor relevant parameters with some of the patients. Home telecare refers to the use of telemedical equipment in the homes of eligible patients, to enable distant monitoring of relevant parameters. Examples are tele-sphygmomanometers,-blood glucose meters, -digital scales, -ECG-devices, and videoconferencing systems.

The AGnES-practice assistant was responsible for the training of the patients who used home telecare, for setting up the devices (e.g. input of necessary data and alarm values in the systems), for controlling a steady and appropriate measuring, data transmission, plausibility and consistency of the data [16].

Optional activities of the AGnES-practice assistants during home visits included geriatric assessment, falls prevention and a detailed assessment of the past and present drug history, including both prescription and over-the-counter-drugs.

The concept of delegating home-visits raised a discussion between organisations and institutions in the healthcare system about feasibility and acceptance of such a concept among physicians and patients.

Therefore, the research questions to be answered in this paper are:

- Which kind of activities are delegated by the GP and conducted by the AGnES-practice assistants during the home visits?

- Do the patients accept home visits, conducted by AGnES-practice assistants?

- Do the patients accept home telecare in a general practice in combination with the AGnES-concept?

- Is the quality of the work of the AGnES-practice assistants, judged by the GPs, and with this, the quality of health care in the AGnES-concept, as good as usual care by a GP?

- Is the implementation of home telecare in a general practice in combination with the AGnES-concept useful?

## Methods

### Qualification and implementation

All participating AGnES-practice assistants were qualified by the project staff and external experts. The qualification included e. g. documentation, communication, knowledge of age-related diseases, pharmaceutics, practical application of falls prevention, practical application of telecare, geriatric assessment [17-19].

The project included three field phases of respectively two, three and five months duration between November 2005 and March 2007. During the first and second field phase, one AGnES-practice assistant (primary qualification: registered nurse) was implemented in a general practice. In the third field phase, two AGnES-practice assistants were implemented in two GP-practices. The study region was the Baltic Sea island of Rügen, belonging to the Federal State of Mecklenburg-Western Pomerania in the very Northeast of Germany (table 2).

**Table 2:** Characteristics of the three field phases

Field phase	1	2	3
Length of the field phase (in months)	2	3	5
Number of AGnES-practice assistants	1	1	2
Number of GPs	1	1	2
Number of participating patients	20	29	56

### Selection of the patients

The GP selected eligible patients to participate in the project, informed them about the concept, obtained informed consent and determined the specific tasks to be performed by the AGnES-practice assistant. Although the GP was free to select the patients, it was achieved to include patients with limited mobility, dependent on home visits.

When a patient was included in the project, the AGnES-practice assistant transferred the patient's personal data as well as his diagnoses, therapy and prescribed medication into the project documentation. Additionally, the patients were asked for information about their household, education and mobility in a base interview. After the different field phases, the patients were asked for acceptance of the concept by completing a final questionnaire.

Use of personal data was restricted to the AGnES-practice assistants and the GP, all scientific monitoring and evaluation was based on pseudonymized data.

### Documentation of the delegated tasks

The AGnES-practice assistants documented all delegated tasks, modules conducted and questionnaire responses into an ergonomically optimized integrated documentation software installed on a portable tablet-pc equipped with touch-screen functionality and character recognition.

Every day, the collected data was encrypted and transferred over a safe connection via the internet (virtual private network, VPN) to the project-server for verification and analysis. Data transfer to the GP was tailored to each physician's individual preferences and included genuine .pdf files and manual entries in the patient files.

### Measurement of the patients' acceptance of the concept

As a measure for acceptance, the patients were asked for their judgement about the concept, the competence of the AGnES-practice assistants in health issues, and if they built confidence in the AGnES-practice assistants.

Upon completion of the second and third field phase (table 2) patients who used telecare devices were asked whether they would be willing to continue using these devices for a longer period of time. In systems with more than one device (e.g. the system digital scale/sphygmomanometer), every device was treated separately. These questions were used as a measure for the acceptance of telecare within the concept.

After every field phase, both AGnES-practice assistants and GPs were asked for a judgement about the usefulness of home telecare in GP-practices.

All questions were assessed by a standardized questionnaire.

### Measurement of the quality of health care

After the second and third field phase (table 2), all participating GPs and AGnES-practice assistants were asked for their judgement about the benefit of the concept and the quality of health care provided to each project patient. The assessment was based on standardized questionnaires.

## Results

### Characteristics of the patients

20 patients participated in the first, 29 in the second, and 56 in the third field phase. Unless otherwise indicated all results refer to the pooled sample of all field studies. 29 patients were male (27.6%), 76 female. The mean age was 73.7, the age range was 37–92 years.

Most patients were multimorbid with chronic diseases including hypertension (75% of the patients), coronary heart disease (55%), diabetes mellitus (52%), cancer (23%), heart failure (7%), and dementia (7%).

In the third field phase, the mobility of each patient was documented in three categories (N = 56): 37.5% of the participating patients were immobile and confined to their beds. 51.8% of the patients were restricted in their mobility and incapable to visit the GP-practice without assistance, 10.7% of the patients were mobile and in principle capable to visit the practice by themselves. In these cases, the GP had arranged the home-visits for other reasons (e.g. temporary immobility, assessment of the home situation and patient's social environment, monitoring of home care).

98.1% of the participating patients (N = 103) completed the base interview, 82.9% (N = 87) completed the final questionnaire. Failure to complete the base interview (N = 2) or the final questionnaire (N = 18) occurred in patients who were physically or cognitively incapable to answer the questions (N = 8), were hospitalized (N = 5), not willing to answer the questions or not at home during the respective period of time when the final questionnaires were applied (N = 7). Hence data analysis is based on 550 home visits with 85 patients, for whom complete data sets were available.

### Implementation of the AGnES-practice assistant: types and content of the home-visits

In the three field studies of the project, a total of 550 home visits were conducted. 98.1% of the participating patients (N = 103) completed the base interview, 82.9% (N = 87) completed the final questionnaire. Failure to complete the base interview (N = 2) or the final questionnaire (N = 18) occurred in patients who were physically or cognitively incapable to answer the questions (N = 8), were hospitalized (N = 5), not willing to answer the questions or not at home during the respective period of time when the final questionnaires were applied (N = 7). Hence data analysis is based on 550 home visits with 85 patients, for whom complete data sets were available.

Table 3 shows the specific activities that were delegated to the AGnES-practice assistants. In the first and second field phase, the GP was hesitant to delegate qualified tasks to the AGnES-practice assistant. Beside the standardized assessment of the patients' health condition, drug history and falls prevention, predominantly monitoring tasks e.g. measuring blood pressure and blood glucose were delegated. During the third field phase the spectrum of delegated tasks was significantly extended mainly with respect to interventional tasks such as conducting complex diagnostic measures and medical counselling.

**Table 3:** Activities delegated to the AGnES-practice assistants

Activities	N
Standardized assessment of the patients' health condition	550
Measuring blood pressure and pulse rate	402
Measuring pulse rate (without measuring blood pressure)	268
Additional documentation of noticeable symptoms	236
Measuring blood glucose	230
Applying standardized tests (e.g. for dementia, mobility)	126
Measuring body weight	81
Drug history (complete ascertainment of medication incl. inspection of storage)	78
Fluid intake advice	76
Training on telemedical devices	58
Standardized prevention of falls incl. visual inspection of the home	45
Measuring performance of the lungs	34
12-lead-ECG	30
Geriatric assessment	26
Instruction how to use a peak flow-meter	18
Drawing of a blood sample	17
Additional advise/consultation (without fluid intake advice)	17
Coordination/check of home care	15
Special talks (e.g. in case of mourning)	15
Checking daily pain journals	13
Therapy of wounds/sores/decubitus	10
Injections	6
Measuring body temperature	5
Issue of a medication plan	2

During 550 home visits, the AGnES-practice assistants initiated 15 unscheduled GP contacts directly from the patients' homes. In 12 cases these were motivated by an unexpected poor state of health of the patient, the remaining 3 contacts referred to problems with or questions about the medication. In all other cases, all information transfer between practice assistant and GP occurred during scheduled contacts.

The mean duration of the home visits was 23 minutes, which is as long as home visits of GPs.

### Patients' acceptance of the AGnES-practice assistants

Most patients (96.5%) felt that the practice assistant was competent for health issues. 93.1% developed a degree of confidence to the AGnES-practice assistant that was comparable to their confidence in their GP. 87.4% of the patients supported the statement that a practice assistant should carry out routine home-visits, prevention and telemedicine, and that GP's home-visits can be restricted to situations requiring special medical attention.

### Implementation of telecare: patients' acceptance und usefulness

During all three field phases, four different telecare-systems were implemented in the homes of a total of 48 (45.7%) eligible project-patients (table 4). The telecare-systems were used over varying time periods ranging from three weeks to four months.

**Table 4:** Implementation of home telecare-systems (N = 48 patients)

Telecare device	N
System digital scale/sphygmomanometer	34
One-lead-ECG	8
System intraocular pressure/Sphygmomanometer/blood glucose meter	4
Twelve-lead-ECG	2

Total	48

The patients were trained by the AGnES-practice assistants in using the various telecare-devices. After the training, most patients had the ability to use the device. In two cases, the wife of the patient provided support, in four cases home care nurses carried out the measurements.

The second and third field phase covered 40 patients with telecare equipment, 17 patients used systems with more than one device, so yielding a total of 57 devices. 75.4% (N = 43) of the patients were willing to continue using telecare devices for a longer period of time. Acceptance depended strongly upon the device. All patients that had used a one-lead-ECG device (N = 4), 78.3% (N = 18) of the patients with a digital scale and 76.9% (N = 20) of the patients with a sphygmomanometer would use this device for a longer time period.

On the other hand, only 2 of 10 patients were willing to use the twelve-lead-ECG equipment for longer times.

All participating GPs and practice assistants judged the implementation of home telecare useful for eligible patients.

### Quality of health care in the AGnES-concept

The participating GPs (one in the second, two in the third field phase) evaluated for 75.3% of the participating patients a particular benefit (high and fair approval combined) from the health care of the AGnES-practice assistant. The participating practice assistants judged 77.6% of the patients to have had a particular benefit (table 5).

**Table 5:** Quality and benefit from medical care during the project, assessed by GPs and AGnES-practice assistants.

**Questionnaire statement**	**"This project-patient had a particular benefit from the medical attendance of the AGnES-practice assistant"**		**"Quality of the AGnES-medical care was at least comparable to quality through common GP-care"**	
**Judgement of**	**GPs** Number of patients (%)	**AGnES-Practice assistants **Number of patients (%)	**GPs** Number of patients (%)	**AGnES-Practice assistants **Number of patients (%)

**No approval**	1 (1.2)	-	1 (1.2)	-
**Little approval**	4 (4.7)	-	2 (2.4)	-
**Partial approval**	10 (11.8)	16 (18.8)	11 (12.9)	2 (2.4)
**Fair approval**	26 (30.6)	15 (17.6)	9 (10.6)	14 (16.5)
**High approval**	38 (44.7)	51 (60.0)	54 (63.5)	58 (68.2)
**Not reported**	6 (7.1)	3 (3.5)	8 (9.4)	11 (12.9)

**Total**	**85 (100)**	**85 (100)**	**85 (100)**	**85 (100)**

The second question addressed the quality of health care provided during the project for each participating patient. The GPs judged the quality of health care during the project, supplied by the practice assistants and the GPs appropriate for 74.1% of the participating patients (high and fair approval combined). The AGnES-practice assistants confirmed this for 84.7% of the patients (table 5).

All participating GPs agreed to the statement, that delegating tasks to a practice assistant relieves them in their daily work.

## Discussion

In this paper we summarize experiences and results from three consecutive field phases of the first AGnES project. The first phase was a small study to test routines and instruments and to evaluate patients' and physicians' acceptance of an AGnES-practice assistant and of home telecare. Over the second and third phase the number and scope of different delegated tasks increased, in parallel with a growing confidence of the participating physicians.

### Kind of activities, delegated by the GP

The physicians' acceptance improved with increasing duration of the project. In the first field phase, the AGnES-practice assistant conducted just the standardized questionnaires relating to the patients' health condition, conducted falls prevention, documented drug history and applied training modules for telecare. During the second and third field phase, many tasks were delegated including conducting ECGs, taking blood samples and providing advice about various themes, including diet and fluid balance.

### Patients' acceptance of home telecare

In the AGnES project, home telecare was accepted very well: 75.4% of the patients could imagine continuing the use of home telecare devices for a longer period of time. Also the AGnES-practice assistants and GPs were positive about implementing telecare equipment in the homes of eligible patients. For the first time in Germany, practice assistants were responsible for training the patients, installation of the devices and controlling the data obtained. The GP received a message only when measurements deviated from the predefined ranges.

Notable are the differences in patients' acceptance between the different telecare devices. The implementation of a twelve-lead-ECG device was not successful: out of 10 trained patients only 2 could handle the particular device effectively. Schwaab et al. [10] reported positive results with the same device. However, the patients in the sample of Schwaab et al. had been preselected from a large group of cardiologic patients and conducted the ECG under controlled circumstances in a hospital. A lower average age (64 years in the sample of Schwaab et al. vs. 73.7 years in our sample) further limits the comparability of Schwaab et al.'s study with ours.

### Quality of health care in the AGnES-concept

After the second and third field phase, the participating physicians judged the quality of health care through a team of GP and practice assistants as appropriate for about three-quarters of the project patients. This corresponds to the results in a systematic literature review of Laurent [20], who concluded that appropriately trained nurses can deliver health care in a comparable quality to primary care physicians.

Rosemann et al. [5] showed that in customary primary care, practice employees are rarely involved in medical tasks in the GP practice. The AGnES project indicated that, with sufficient qualification, practice assistants have the potential to be integrated in genuine medical tasks such as home visits.

All participating GPs reported that delegating specific tasks to practice assistants supported them in their daily work. Notable is that all participating GPs planned to continue the cooperation with an AGnES-practice assistant in some form, for example by engaging the AGnES-practice assistant further after the project or by increasing the number of working hours of qualified practice staff for conducting home visits.

### Implementation of home telecare in a general practice

Both participating GPs judged the implementation of telecare-devices to support patient-monitoring useful. An important requirement here is that there is no additional workload for the GP. In the project, the AGnES-practice assistants were responsible for handling the telecare part. The GPs were only involved selecting the patients and assessing alarm values. This way of handling with telecare in a GP-practice was successful.

### Limitations

Our study has some limitations. Due to its design as a proof of concept, the number of participating GPs and AGnES-practice assistants is too small to allow for meaningful evaluation of subgroups. Also, the duration of the field studies was short (2–5 months), limiting the possibility to measure longterm clinical results in the patients who participated.

### Further research

Based upon the experiences in this project, new projects in different settings with more GPs and AGnES-practice assistants have been initiated in four federal states in Germany. In the Federal State of Brandenburg, a project started in July 2006 with three practice assistants (all registered nurses), supporting six GPs in a regional medical centre. In the Free State of Saxony, six AGnES-practice assistants (three registered nurses and three physician's assistants) support six GPs in underserved areas since July 2007. In the Federal State of Mecklenburg-West Pomerania a new project started in July 2007. Here, three AGnES-practice assistants (two registered nurses and one physician's assistant) support 14 GPs in three regions. In December 2007, a project with 8 AGnES-practice assistants in 4 GP-practices was launched in Saxony-Anhalt. A corresponding project investigates legal aspects of qualification and training as well as limits to the delegation of physicians' tasks to practice assistants.

### Perspectives

The projects received considerable media coverage and triggered a broad discussion about structural challenges for GPs in rural regions and new concepts to delegate medical tasks and competences to the different professions in health care.

The AGnES projects demonstrate considerable potential to initiate changes in the health care system in Germany. Every participating federal state initiated a steering committee representing major institutions in health care in order to facilitate and accelerate the transfer of the project experiences and results into the health care system. Additionally, delegates of the ministries of health of all eastern German federal states initiated an "AGnES-working group".

In three of the subsequent projects (in Saxony, Saxony-Anhalt and Mecklenburg-Western Pomerania), two important partners, the Association of Statutory Health Insurance Physicians and the statuatory insurances themselves, are cooperating both conceptually and actively contribute to the funding of AGnES projects.

As a result of the activities of the "AGnES-working group", in March 2008, a change in legislation made delegation of home visits to AGnES-practice assistants possible. At the moment, representatives of the federal Kassenärztliche Vereinigung and the statuatory insurances are negotiating the terms of reimbursement of these delegated home visits.

## Conclusion

This project has shown that the model of delegation of home visits to qualified staff in GP practices is a way to support GPs in regions with an imminent or already existing undersupply with GPs. AGnES-practice assistants have potential to perform a variety of tasks in primary medical care. More research is necessary to specify the required qualification. In further projects, clinical parameters of the patients will be analysed to assess the quality of medical care in this concept in a more objective way.

Politically, the AGnES project has helped initiating a discussion how to integrate other medical professions in primary care. Delegation may help to meet the challenge to maintain quality of medical care in rural regions in Germany.

## Competing interests

The authors declare that they have no competing interests.

## Authors' contributions

WH and NvdB conceived the study design. NvdB drafted the manuscript. NvdB, TF, CM, RH, performed the study and analysed the data. SS participated in developing the concept. All authors contributed substantially to the manuscript. All authors read and approved the final manuscript.

## Pre-publication history

The pre-publication history for this paper can be accessed here:



## References

[B1] Statistisches Bundesamt (2003). The German population from 2002 until 2050 – 10. coordinated population prognosis [Bevölkerung Deutschlands von 2002 bis 2050–10. koordinierte Bevölkerungsvorausrechnung].

[B2] Statistisches Landesamt Mecklenburg-Vorpommern (2003). 3. prognosis of the development of the population of Mecklenburg-Western Pomerania [3. Landesprognose Bevölkerungsentwicklung in Mecklenburg-Vorpommern bis 2020].

[B3] Fendrich K, Hoffmann W (2007). More than just aging societies: the demographic change has an impact on actual numbers of patients. J Public Health.

[B4] Klose J, Uhlemann T, Gutschmidt S (2003). Lack or Surplus of Physicians? – Effects of Aging of Physicians on Medical Care [Ärztemangel – Ärzteschwemme? – Auswirkungen der Altersstruktur von Ärzten auf die vertragsärztliche Versorgung].

[B5] Rosemann T, Joest K, Körner T, Schaefert R, Heiderhoff M, Szecsenyi J (2006). How can the practice nurse be more involved in the care of the chronically ill? The perspectives of GPs, patients and practice nurses. BMC Family Practice.

[B6] Carter YH, Shaw S, Macfarlane F (2002). Primary Care Research Team Assessment (PCRTA): development and evaluation. Occas Pap R Coll Gen Pract.

[B7] Bridges J, Fitzgerald L, Meyer J (2007). New workforce roles in health care: exploring the longerterm journey of organisational innovations. J Health Organ Manag.

[B8] Porzsolt F, Ghosh AK, Kaplan RM (2009). Qualitative assessment of innovations in healthcare provision. BMC Health Services Research.

[B9] Celler BG, Lovell NH, Chan DK (1999). The potential impact of home telecare on clinical practice. Med J Aust.

[B10] Schwaab B, Katalinic A, Richardt G (2006). Validation of 12-lead tele-electrocardiogram transmission in the real-life scenario of acute coronary syndrome. J Telemed Telecare.

[B11] Alte D, Völzke H, Robinson DM (2006). Tele-electrocardiography in the epidemiological 'Study of Health in Pomerania' (SHIP). J Telemed Telecare.

[B12] Ladyzyński P, Wójcicki JM (2007). Home telecare during intensive insulin treatment – metabolic control does not improve as much as expected. J Telemed Telecare.

[B13] Eberl R, Biskup K, Reckwitz N (2005). The televisit system in patients care after discharge in clinical use – first experiences [Das System der Televisite zur poststationären Patientenbetreuung im klinischen Einsatz – Erste Erfahrungen]. Biomed Technik.

[B14] Kiefer S, Gersonde F, Klusen K, Meusch A (2002). Homecare networks, using the example of aftercare of stroke patients [Homecare-Netzwerke am Beispiel Schlaganfall-Patientennachsorge]. Gesundheitstelematik – Medizinischer Fortschritt durch Informationstechnologien.

[B15] Macduff C, West B, Harvey S (2001). Telemedicine in rural care part 1: developing and evaluating a nurse-led initiative. Nursing Standard.

[B16] Terschüren C, Fendrich K, Berg N van den (2007). Implementing telemonitoring in the daily routine of a GP practice in a rural setting in northern Germany. J Telemed Telecare.

[B17] Kalbe E, Kessler J, Calabrese P (2004). DemTect: a new, sensitive cognitive screening test to support the diagnosis of mild cognitive impairment and early dementia. Int J Geriatr Psychiatry.

[B18] Sunderland T, Hill JL, Mellow AM (1989). Clock drawing in Alzheimer's disease. A novel measure of dementia severity. J Am Geriatr Soc.

[B19] Podsiadlo D, Richardson S (1991). The timed "Up & Go": a test of basic functional mobility for frail elderly persons. J Am Geriatr Soc.

[B20] Laurant M, Reeves D, Hermens R (2005). Substitution of doctors by nurses in primary care. Cochrane Database Syst Rev.

